# Exploring the status of the human operator in Industry 4.0: A systematic review

**DOI:** 10.3389/fpsyg.2022.889129

**Published:** 2022-09-20

**Authors:** Liliana Cunha, Daniel Silva, Sarah Maggioli

**Affiliations:** ^1^Faculty of Psychology and Educational Sciences of the University of Porto, Porto, Portugal; ^2^Center for Psychology at University of Porto (CPUP), Porto, Portugal

**Keywords:** Operator 4.0, Industry 4.0, technological transformations, human-centered approach, emerging risks, work sustainability

## Abstract

Industry 4.0 (I4.0) promises to transform jobs and working conditions through the implementation of unprecedented human-machine interaction modes. As the operator working in these new settings, known as the Operator 4.0, is a relatively recent concept, and although technological developments are expected to support workers and require higher labor skills, the risks and health impacts resulting from these changes remain underexplored. This systematic review aims to (i) systematize literature findings on how workers are perceived and participate in I4.0 work systems; (ii) identify the main technological changes driven by I4.0; and (iii) instigate discussion regarding the impacts these changes may have for workers and the sustainability of work systems. Following a systematic review approach using the PRISMA protocol, the articles were organized into two main analysis axes: the technical changes brought about by I4.0, and the representation of the human worker within these new work settings. The findings reveal that a techno-centered approach still seems to be dominant in guiding the implementation of I4.0 models; secondly, as a consequence, the social dimensions of work tend to remain as residual issues, overshadowed by the promises related with technology (e.g., productivity, efficiency); finally, the representation of the Operator 4.0 remains blurry, as he/she is perceived as gender neutral, skillful, and perfectly fit for work, assuring the functioning (and compensating for the limits) of these systems. While I4.0 promises safer and more productive workplaces, issues related to employment conditions, emerging risks and health impacts become more prominent when analyzed from an activity-centered perspective. In terms of future research, a more heuristic analysis could be achieved through a participatory and work-centered approach and following a gender perspective. This way, visibility could be conferred to another side of I4.0, thus guaranteeing conditions for the sustainable development of these work situations.

## Introduction

The growing implementation of digital and automation technologies in the industrial context, including collaborative robots (cobots), algorithms, artificial intelligence, Internet of Things (IoT), Big Data, and Cyber-Physical Systems (CPS), integrate a new paradigm known as the Fourth Industrial Revolution (4IR), also named Industry 4.0 (I4.0), Factories of the Future (FoF), or even Smart Manufacturing (Iordache, 2017; Gualtieri et al., 2020; Kadir and Broberg, 2021). Within this movement, interconnected intelligent factories are envisaged to allow efficient data collection and processing, distributing and guiding operations in an automated way while also allowing operational monitoring in real time (Moro et al., 2019; Çinar et al., 2021). Thus, this type of technological development is presented as giving companies a competitive advantage, in which some jobs more than others, especially those involving “repetitive tasks,” are expected to be more susceptible to replacement by automation (e.g., Frey and Osborne, 2017), improving productivity through collaborative work between people and machines (Stern and Becker, 2019; Broday, 2020).

Equipped with these new technologies, the operators who participate in these systems will interact with a plethora of technical innovations in performing their activities. In this context, the human operator – designated as the “Operator 4.0” – tends to be typified according to the technologies used. For instance, when using exoskeletons, the worker is depicted as a super-strength operator, or a healthy one when using smart wearable solutions which collect psychophysiological data (Romero et al., 2016a; Ruppert et al., 2018). From this point of view, some authors underlined new technologies, principally exoskeletons (body-worn assistive devices) and cobots, have the potential to improve productivity and occupational health (e.g., prevention of musculoskeletal disorders by reducing the load on the muscular system) (Cimini et al., 2020; Ranavolo et al., 2021). Still, implementing such technologies does not assure, *per se*, that the risk of musculoskeletal issues will be reduced, as Cockburn (2021) and Bounouar et al. (2022) have recently stressed. Additionally, a few authors have claimed that, by relieving workers from repetitive and monotonous tasks, technology could support them in improving their skills, particularly related to the supervision of the work system. In this sense, workers may be expected to be more qualified and more autonomous (Romero et al., 2016a; Thun et al., 2019; Broday, 2020). Other voices, however, considered the possibility of these new technologies contributing toward the increase of control over employees (e.g., through new sensoring and monitoring applications); work intensification; gender segregation; and, at the same time, narrowing the margin workers have for decision-making (Piasna and Drahokoupil, 2017; Moro et al., 2019; Beer and Mulder, 2020; Kaasinen et al., 2020; Kadir and Broberg, 2020; Golsch and Seegers, 2021).

As for the health consequences that could potentially stem from I4.0, some impacts have been identified in literature, such as mental health issues (associated with the reduction of autonomy and increased skill requirements), others associated with the use of tangible automation technologies (e.g., cobots and automated vehicles leading to an increased cognitive workload), or even the feeling of job insecurity due to an increased incorporation of technology (Golsch and Seegers, 2021; Kadir and Broberg, 2021; Reiman et al., 2021). However, the actual extent of the impacts these technology-enabled changes have on workers' health and their activities remains as an “uncharted territory” (Badri et al., 2018; EU-OSHA, 2018; Bobillier Chaumon, 2021; Zorzenon et al., 2022).

In tune with the concept of I4.0 as a “new industrial stage,” triggered by the implementation of a set of emerging technologies in order to “modernize” and increase the whole productivity cycle (Frank et al., 2019), up to now research seems to be more concentrated on the technical aspects associated with the I4.0 transformations rather than on human work. Concretely, Neumann et al. (2021), Barcellini et al. (2021), and Bentley et al. (2021) debated that in the context of 4IR, attention to human aspects has been particularly sparse. What is more, at issue is, as Bentley et al. (2021) stressed, the risk of designing future work situations taking into account a single work system component, i.e., technology. By concentrating on technological push-factors to achieve new levels of productivity and health, decision-makers tend to focus on the potential of technology and end up sidelining the social dimension of the I4.0 workplace (Moniz and Krings, 2016; Neumann et al., 2021; Pacaux-Lemoine et al., 2022). This includes working conditions, new models of work organization, the ways workers interact with technology, the new sources of constraints and resources introduced by technology, the changing skill requirements, opportunities for learning, or the emerging risks that could threaten workers' health and wellbeing (Barcellini, 2019; Bounouar et al., 2022). As reflected by Moniz and Krings (2016), these issues tend to be viewed only from the perspective of technical improvements and safety (in terms of the interaction between the worker and technology). For example, Galey et al. (2021) added that some approaches seek to make innovations safer by focusing primarily on technical dimensions or without considering the actual work and potential risks associated to those introductions. Such “techno-centered” depictions seem to leave little room to explore how work and its underlying conditions are actually reconfigured by I4.0 technologies, and the status of human operators within these work environments. This suggests, as alluded to by Neumann et al. (2021), that the I4.0 research is somehow “blind to the nature of the human-system interactions” (p. 5) in the systems they are frequently required to help design. As a consequence, – the authors continue – “this does not bode well for the success of I4.0 approaches, or for the people forced to endure them” (Neumann et al., 2021, p. 5).

Although techno-centered literature reviews have been covered regarding the implementation of I4.0, there is still the need to understand these transformations from other perspectives (Nayernia et al., 2021). Concretely, from the point of view of the operators and their work activities. Hence, this review addresses, on the one hand, the main workplace transformations circumscribed in the I4.0 models. And, on the other hand, it explores the characterization of the status assumed for the Operator 4.0 the risks which emerge from them, and the impacts on health. Thus, our review pursues the following research questions:

RQ1. What workplace transformations from I4.0 are expected?
RQ2. How is the Operator 4.0 represented, considering the skills needed to work in these contexts, and what risks could he/she be exposed to?

Through this review, gaps in the literature are identified and discussed, while providing some directions for future work.

## Methods

### Search strategy

The research presented in this article followed a systematic approach using the preferred reporting items for systematic review and meta-analysis protocols (PRISMA) methodology. Therefore, it defines a clear aim which is addressed in a repeatable and thorough manner (see Shamseer et al., 2015). For the research, the cross-disciplinary database Scopus was used, as it is the main database for peer-reviewed publications. This choice was made taking into account that Scopus covers the widest range of indexed journals (e.g., Falagas et al., 2008). The search terms used for this review were: “Operator 4.0”; “Factories/Factory of the future,” “Industry 4.0,” and “Smart operator.” Articles were collected between September and November of 2021. Given the emergent nature of the concepts under analysis, only articles from the last 5 years were included. That is, despite the I4.0 paradigm having emerged ~10 years ago, in the last 5 years there has been an intensification of the literature published on the topic (Liao et al., 2017). Also, as it was 5 years ago that the European Commission's Horizon 2020 project specifically dedicated to the “Factories of the Future” was established (European Commission, 2016), the publication year filter allowed for publications regarding real workplace implementations of this type of technologies to be prioritized over merely test-type applications.

The terms were searched in abstracts, titles, and keywords, with the following inclusion criteria (see Table 1): publication year (published from and including 2015), their scientific domain (in this case, only articles under the “Social Sciences” category were included when the number of articles allowed this filter to be selected) and language (English). This subject area was selected considering that it is the area in which our scientific tradition fits in. Assuming this “human” is always a being in activity, in a given context and with a professional history, these dimensions always influence the relationship developed with the technical objects. For this reason, purely technical areas of research were excluded.

**Table 1:** Keywords and filters used in the research.

**KEYWORD**		**PUBYEAR**		**LANGUAGE**		**SUBJAREA**
Operator 4.0 AND “Operator 4.0”	AND	> 2014	AND	“English”	AND	“SOCI”
“Industry 4.0”	AND	> 2014	AND	“English”	AND	“SOCI”
“Factory of the future” AND “Factories of the future”	AND	> 2014	AND	“English”	AND	“SOCI”
“Smart operator”	AND	> 2014	AND	“English”	^*^	^*^

* For this keyword, no articles were found under the “Social Sciences” field filter.

Once the relevant articles were collected, filtered and the duplicates removed, the papers were once again selected with a view of eliminating those that were not directly related to our objectives, resulting in a total of 77 papers.

Regarding the data analysis, at an initial stage all the authors reviewed the meta information of each article: title, abstract, and type of publication. Based on the full content of each selected paper, the data extraction was performed focusing on our research objectives. To reduce research bias, the authors consistently held meetings to ensure agreement regarding the distribution of the articles according to the specific research question. Then, following an inductive approach, the selected papers were distributed throughout the three main topics. A database was created to summarize the main information extracted from each article, which supported the tabulation of the results presented in the next section. In addition, the discussion of the results also included the contribution of other papers which are part of the epistemological framework of the scientific tradition of work psychology and activity ergonomics.

## Results

**Figure 1:**
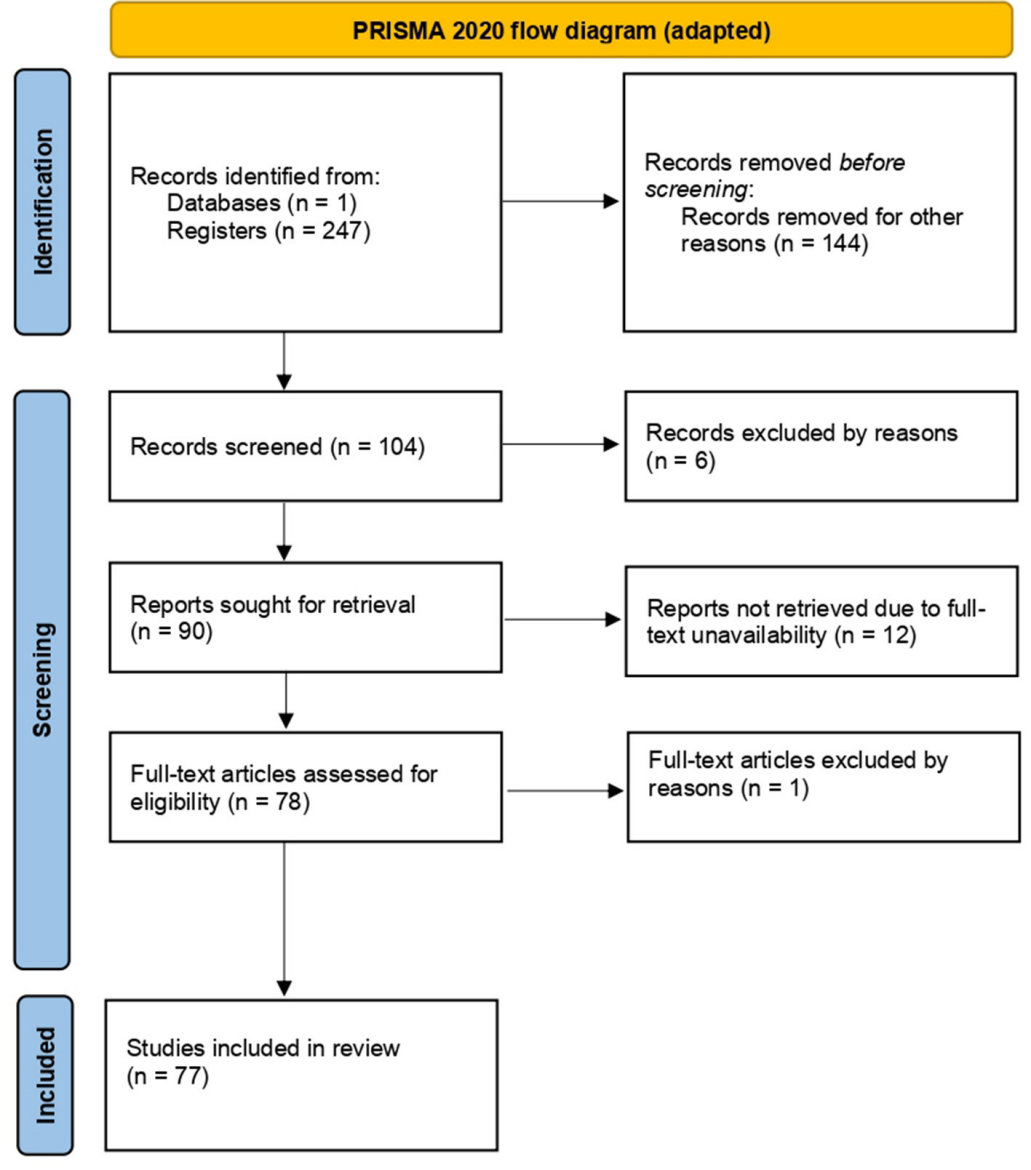
Research and reference selection process flowchart using the preferred reporting items for systematic review and meta-analysis protocols (PRISMA).

The process followed during this review to gather literature and the structure which led to the final selection of articles is illustrated in Figure 1.

**Figure 2:**
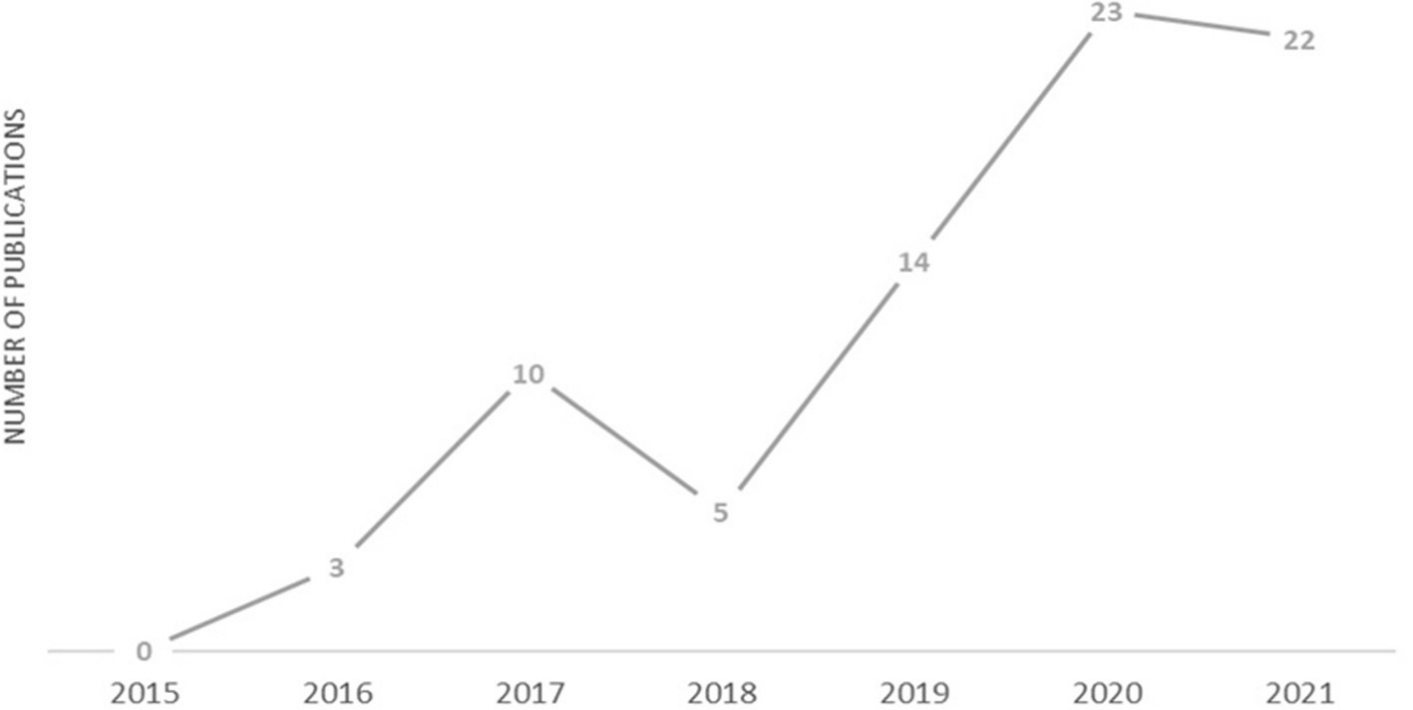
Distribution of the filtered publications included in the analysis per year since 2015.

Figure 2 illustrates how the final selection of articles are distributed throughout the last years.

The results described in the following sections are all based on the articles relating to the theme in analysis. Table 2 synthetizes how the obtained results articulate with our research questions.

**Table 2:** Distribution of the selected articles according to the research questions.

**Research question**	**Corresponding topic**	**Topic content description**	**Authors**
RQ1: What workplace transformations from I4.0 are expected?	Topic 1 Industry 4.0: transformations, contrasting visions and challenges for implementation	1.1. The I4.0 context was characterized through the description of the main concepts, objectives and challenges of its implementation from a more macro point of view (e.g., business model analysis; theoretical frameworks; system efficiency assessment)	Richert et al., 2016a; Gregor et al., 2017; Iordache, 2017; Lee et al., 2017; Santos et al., 2017; Badri et al., 2018; Um et al., 2018; Gray-Hawkins et al., 2019; Hamdi et al., 2019; Kumar and Kumar, 2019; Madonna et al., 2019; Ahmad et al., 2020; Blštáková et al., 2020; Brozzi et al., 2020; Cimini et al., 2020; Gajšek et al., 2020; Gazzaneo et al., 2020; Longo et al., 2020; Miśkiewicz and Wolniak, 2020; Ramadan et al., 2020; Berrah et al., 2021; Çinar et al., 2021; El-Haouzi et al., 2021; Rupp et al., 2021; Stawiarska et al., 2021
		1.2. I4.0 technologies were described through case studies on specific components, which included both implementation and trial studies, as well as their expected outcomes and limits.	Richert et al., 2016b; Kolbeinsson et al., 2017; Langfinger et al., 2017; Thomay et al., 2018; Ivaschenko et al., 2019; Minnetti et al., 2019; Pavel et al., 2019; Baldissone et al., 2020; Digiesi et al., 2020; Fruggiero et al., 2020; Gualtieri et al., 2020; Kadir and Broberg, 2020; Santo et al., 2020; Shi et al., 2020; Van Acker et al., 2020; Agnusdei et al., 2021.
RQ2: How is the Operator 4.0 represented, considering the skills needed to work in these contexts, and what risks could he/she be exposed to?	Topic 2 The status of human work in I4.0	The representation of the new operator 4.0 was analyzed, as well as the skills which are expected from him/her, and how they are considered, involved, and integrated into the new work systems. The main challenges for employment and workplace training in I4.0 were also taken into account when discussing the role workers play in these contexts.	Kolbeinsson et al., 2017; Marrella and Mecella, 2017; Schloegl et al., 2017; Badri et al., 2018; Mark et al., 2019; Thun et al., 2019; Udayangani et al., 2019; Ahmad et al., 2020; Blštáková et al., 2020; Broday, 2020; Cimini et al., 2020; Gajšek et al., 2020; Gazzaneo et al., 2020; Gualtieri et al., 2020; Hoyer et al., 2020; Kaasinen et al., 2020; Kadir and Broberg, 2020, 2021; Longo et al., 2020; Saabye et al., 2020; Sony and Naik, 2020; Chistyakova et al., 2021; Di Carlo et al., 2021; Golsch and Seegers, 2021; Ivaldi et al., 2021; Paliga and Pollak, 2021; Patriarca et al., 2021; Rangraz and Pareto, 2021; Reiman et al., 2021; Shi et al., 2021; Tortora et al., 2021.
	Topic 3 Risks and impacts on health: Innovation, but at what cost?	The main emerging risks and impacts on health in the context of I4.0 were discussed.	Ansari et al., 2018; Adriaensen et al., 2019; Gunasekaran, 2019; Blštáková et al., 2020; Fruggiero et al., 2020; Hoedt et al., 2020; Longo et al., 2020; Saabye et al., 2020; Serras et al., 2020; Rangraz and Pareto, 2021; Weiss et al., 2021.

Taking into account our two research questions (RQ1 and RQ2), information regarding three main topics was extracted. In the case of Topic 1, the literature review process was further broken down into subtopics to synthetize and report the findings (Petticrew and Roberts, 2006). Considering our RQ1, this option is due to the fact that this topic resulted from the attempt to explore the different types of workplace transformations (e.g., automation, process digitalization, robot implementation) which are expected to take place with the development of I4.0 technologies. Given the heterogeneity in terms of research designs and the contributions in the studies which were found, ranging from macro to micro points of view on how I4.0 can impact workplaces, the reviewed articles were categorized into two subtopics considering their scope and their distance from the human work developed on a day-to-day basis in a given context. Thus, on the one hand, studies contributing to conceptualizing I4.0 through theoretical frameworks, large scale transformations, new business models, or which analyzed production processes as a whole, were grouped into the first subtopic (1.1) of Topic 1. On the other hand, studies using a methodological approach based on case studies, including concrete implementations or trials were coded as belonging to the second subtopic (1.2). From both levels of analysis, the impacts of these transformations were considered. Therefore, the section 4.1 of the Discussion resulted in the description of technologies (e.g., cobots, artificial intelligence, sensors) and their underlying concepts, considering the approaches which guide them (techno-centered or human-centered), thus enabling the understanding of the work contexts from which the concept of Operator 4.0 emerges, a representation addressed in RQ2. Subsequently, this concept is discussed considering the use of the technology previously explored and how the process of its implementation takes place (see Topic 2). Lastly, in Topic 3, risks and impacts for health were explored, as well as how the sustainability of human work in these scenarios is threatened (answering the second part of our RQ2).

## Discussion

### Industry 4.0: Transformations, contrasting visions and challenges for implementation

The concept of Industry 4.0 originated in Germany and focused on the digitalization of manufacturing processes (Çinar et al., 2021). The technologies involved enable the interconnection of different sectors, actors, systems, and artifacts, creating intelligent manufacturing systems, and changing the focus of mass production into mass customization (Um et al., 2018; Gualtieri et al., 2020; Ramadan et al., 2020; Agnusdei et al., 2021; El-Haouzi et al., 2021). This concept encompasses a series of technological components which is briefly synthesized and listed in Table 3.

**Table 3:** Synthesis of the main Industry 4.0 related concepts.

**Main concepts**	**Brief definitions**
Industry 4.0 (or I4.0)	The movement which converges manufacturing with the digital revolution, corresponding to what is considered to be the fourth industrial revolution, emphasizing cooperation between industry and science, and, consequently, between knowledge and skills (Iordache, 2017; Badri et al., 2018; Rupp et al., 2021). It consists of a network with embedded electronic devices, allowing the collection and exchanging of data (Santos et al., 2017).
Factories of the Future (or FoF)	Factories which allow efficient data collection and processing, while also allowing operational monitoring in real time (Moro et al., 2019; Çinar et al., 2021). They are also defined as “Context-aware” factories, assisting people and machines in the execution of their tasks, being able to “communicate and interact with the environment” (Lee et al., 2017, p. 2–3).
Smart factory	Intelligent interconnected factories, able to react to changes, adapt to different manufacturing processes and interact with the different actors present in the system, assisting both people and machines in their tasks (Lee et al., 2017; Miśkiewicz and Wolniak, 2020). They do so by being able to aggregate data from sensors through a network system (Lee et al., 2017). Through collaborative robots and exoskeletons (Longo et al., 2020), the main goal of the smart factory is to make complex structures in manufacturing processes more accessible and manageable (Shi et al., 2020).
Smart systems	Systems which allow objects to control and communicate inside themselves and with their surroundings due to real-time access to information, controlled production, just-in-time supply, and autonomously process data based on self-managing computer systems, which are self-configuring, self-protecting, self-healing, self-optimizing, which process and analyze Big Data (Ansaldi et al., 2018; Miśkiewicz and Wolniak, 2020).
Digital twin	Virtual representatives of the real objects (or the whole factory) which exist in addition to the real objects and are created to improve the efficiency and profitability of Industry 4.0 systems as they can predict risks and/or anomalies and communicate with a server to generate a warning before it happens (Gregor et al., 2017; Agnusdei et al., 2021).
Augmented reality	Interface which enables the operator to, through virtual information, access and view the real environment *via* technology (Langfinger et al., 2017; Baldissone et al., 2020; Gajšek et al., 2020). It can facilitate decision-making processes as it allows the development of interactive user interfaces (Langfinger et al., 2017; Ivaschenko et al., 2019).
Cyber-physical systems (or CPSs)	Systems in which computerized elements collaborate to monitor and control physical entities, as they are connected to the physical world, but allow data accessing and usage (Badri et al., 2018; Berrah et al., 2021). Kumar and Kumar (2019) describe these systems as the core technology of I4.0, because they can access information regarding the environment using sensors, but it can also share the information through the network and enable real-time action (Gregor et al., 2017).
Collaborative robots (or cobots)	Robots which have the capability to work with humans in these manufacturing environments, aiding them in their needs, based on combining information sciences, human factors, biomechanics and robotics (Ansari et al., 2018; Badri et al., 2018; Ahmad et al., 2020; Romero et al., 2020). According to Ahmad et al. (2020), the main benefit of this kind of technology is that it can reduce costs and improve the automation of manufacturing/production and management processes.
Smart wearable solutions	Devices which monitor in real-time a range of Operator 4.0 vital signs and the surrounding workplace environment, through sensors and ambient intelligence, and can thus help to make sure operators are healthy and safe (Gazzaneo et al., 2020; Romero et al., 2020). They are also enhancers of the operator's sensorial and interaction capabilities (Um et al., 2018; Gazzaneo et al., 2020).
Artificial intelligence	Theories, techniques, and technologies developed in order to develop machines capable of simulating intelligence (Badri et al., 2018).
Cloud computing/cloud solutions	New Information Technology (IT) services model which works using the internet, where the IT functionalities are offered as an external service and allow data storage (Gregor et al., 2017).
Internet of things	The platform where all production systems become interconnected, as exchanges of information and data coming into it from devices performing real tasks in the physical world (Badri et al., 2018; Miśkiewicz and Wolniak, 2020). It allows the access to data from anywhere and the exchange of data between devices, that is, guaranteeing interoperability (Miśkiewicz and Wolniak, 2020).
Big data	Large datasets that surpass “human intuitive and analytical capacities and even those of conventional computing tools for database and information management” (Badri et al., 2018, p. 405). This data is produced from a large range of sensors, technological artifacts and social media, and are transmitted over the Internet to then be stored in the previously mentioned cloud solutions (Ahmad et al., 2020). In Industry 4.0 contexts data is generated by several sources like machine controllers, sensors, manufacturing systems, people, among many others. All this voluminous data, arriving at high velocity and in different formats is called “Big Data.” The processing of Big Data in order to identify useful insights, patterns or models is the key to sustainable innovation within an Industry 4.0 factory (Santos et al., 2017)

However, the focus of this analysis was mainly to explore their underlying approaches and to understand how the human-technology relationship has been constructed from different points of view. When examining technological changes, it is not uncommon for these to be described in a neutral way, regarding their relation to the workers and contexts which shape them. This new vision of industry brought by I4.0 promises to improve performance, make manufacturing more effective, customizable, and easily manageable, with a resulting increase in productivity which gives companies a competitive advantage (Kolbeinsson et al., 2017; Minnetti et al., 2019; Kadir and Broberg, 2020; Santo et al., 2020). That is, technology is conceptualized almost as if it carries all the potential for success and could just function by itself. Hence, from a critical viewpoint, the techno-centered and human-centered approaches were contrasted to discuss these reconfigurations, considering that it is the human interaction that makes the new technological systems reliable, as will be further explored ahead.

Therefore, in light of our first research question (RQ1), the technological components described in Table 3 are what allow the main principles of I4.0 to be outlined: interoperability, real-time access to data, virtualization, decentralized decision-making, and demand/service-oriented production (Santos et al., 2017). Generally speaking, people, machine, equipment, logistics systems and components are expected to communicate and cooperate with each other as these factories become adaptable and flexible (Iordache, 2017; Gray-Hawkins et al., 2019; Gajšek et al., 2020). Following this perspective and in contrast to previous work scenarios, new forms of human-machine interaction are supposed to assist workers safely and efficiently, in a diverse range of environments and tasks, even when these are highly dynamic and uncertain (Richert et al., 2016a). In this sense, artificial intelligence algorithms are expected to play a significant role in optimizing production processes (Kumar and Kumar, 2019).

In order to support the discussion which takes place throughout this article, it is thus relevant to define the two main perspectives that guide the design of industrial 4.0 systems: the techno-centered approach and the human-centered one. The first one consists of a perspective focused on the optimization of industrial production systems, with priority being given to its technical components, as critically remarked by Colim et al. (2020). This approach tends to overestimate technology, assuming that the worker will ensure the supervision of the entire system while not properly describing how this can be guaranteed (Trentesaux and Millot, 2016; Pacaux-Lemoine et al., 2017; Ngoc et al., 2022). Often, it assumes a representation of the human factor as an omnipresent operator, who will manage all non-automatable problems and events which are impossible to anticipate and ensure the recovery of production after an unforeseen event. Therefore, this operator is seen as someone who intervenes (only) when necessary, with a timely adequate response, and who always has a consistent response to work demands, regardless of the variation of his/her circadian level along the day, or even of his/her level of accumulated fatigue (Trentesaux and Millot, 2016). The human operator therefore ends up having the status of an “adjustment variable” for the functioning of such complex systems, integrated in contexts that leave little room for debate about the difficulties posed by new technologies. However, even when the focus is primarily given to technology through this type of vision, issues regarding the functioning of technology occur, as its application is influenced by the conditions found in the different work contexts. Badri et al. (2018) highlighted issues related to calibration, data, network, and artificial intelligence reliability, the absence of standards for the interaction with cobots, and personal data confidentiality.

In contrast, the human-centered approach, also called “anthropocentric,” links sociotechnical changes with operators and organizational decision-makers, in a comprehensive approach which involves both the pre-existing and reconfigured forms of work activity, as well as the system that structures it (Béguin and Cerf, 2004; Barcellini et al., 2015; Bobillier Chaumon et al., 2019). This perspective is anchored in the analysis of the real work activity and its reference situations (e.g., Galey et al., 2021), in order to anticipate the future forms of activity in the emerging environments of I4.0 (Barcellini, 2020; Galey et al., 2020). According to May et al. (2015), the design of the workplaces of the future must favor this type of view over a purely technological one, placing the worker at the center of the system as an active agent in its optimization. The importance of the adoption of this view has gained more visibility and can be found under the standard ISO 9241-210:2019 (Berrah et al., 2021; El-Haouzi et al., 2021), which defines it as “A way of designing interactive systems, aiming to make systems usable and useful by focusing on users, their needs and requirements, and applying human factors, ergonomics and existing knowledge and techniques in terms of usability” (ISO 9242-210:2019 in El-Haouzi et al., 2021, p. 2). Also, the European Factories of the Future Research Association (EFFRA), for example, invoked the human-centered perspective as a requirement for the development of FoF (EFFRA - European Factories of the Future Research Association, 2016).

Possibly due to the fact that I4.0 is still a process under development and to the lack of consensus between these two main contrasting perspectives which guide the way research is conducted, there are still conceptual issues which need to be tackled. For instance, there are no clear common guidelines about how the new Operator 4.0 is involved in these complex work systems (Gazzaneo et al., 2020). Kumar and Kumar (2019) mentioned that human cognitive workload had still not been considered in industrial practices. Two years later, Tortora et al. (2021) highlighted that even though experience and training have been identified as the most critical human factors for performance, research still had not considered them. However, even in the studies developed by Thomay et al. (2018), Madonna et al. (2019), Digiesi et al. (2020), Cimini et al. (2020), and Van Acker et al. (2020), the cognitive workload is included but focused mainly on performance, evaluated through physical parameters and task sequences, with the aim of evaluating the so-called “human errors.” In the case of the latter study, the use of these measures is argued as “relevant for situations in which the consequences of hesitative behavior can be detrimental” (Van Acker et al., 2020, p. 35). But in real working conditions, and in industrial production settings in which productivity continues to be the main focus, will there be the guarantee that work is interrupted when the cognitive workload affects workers' health? This question remains open, as does the research on the social dimensions of I4.0 (e.g., El-Haouzi et al., 2021), in which no actual framework or definition of methodological paths have been described. These limitations can have further consequences for the technology's applicability (Shi et al., 2020).

Also contradicting the vision of perfectly adapted factories is the idea that technology is supposed to become an extension of the human in the center of these systems - as mentioned, for example, by El-Haouzi et al. (2021) -, but authors still mention the need to integrate workers into I4.0 settings and not the other way round (e.g., Cimini et al., 2020; El-Haouzi et al., 2021). Santos et al. (2017), Pavel et al. (2019), and Hamdi et al. (2019) defined I4.0 contexts but did not mention the workers of these contexts even once. Gregor et al. (2017) described logistics and smart factory functioning, but also did not make any reference to workers or consider them in their analysis. In another article developed by Fruggiero et al. (2020), reflection on the importance of considering humans in these work systems and of taking into account risk factors (such as psychosocial ones) was developed. Nevertheless, the perspective that the worker is the one who needs to adapt to work was still assumed: “the cooperation between human and robot can gain in reducing the workers load while increasing capacity. In this case it is required a correct assignment of the humans to the task” (Fruggiero et al., 2020, p. 591).

Bearing this in mind, can we really consider technology to be adapted to work situations and their protagonists and not the opposite? Up to now, research seems to contradict the human-centered approach. Therefore, what is under debate is the conceptualization of the Operator 4.0 and the status conferred to him/her in these work scenarios.

### The Operator 4.0 concept and the status of human work in I4.0

Even though the industrial transformations under analysis predict the development of logistic automation and self-management components, a human presence remains fundamental. Rather than being replaced by technology, the focus of I4.0 lies, according to Cimini et al. (2020) and Paliga and Pollak (2021), in relieving these workers from strenuous and monotonous tasks, and also in developing other skills allowing workers to be supported in the management of these new and complex systems. The role this worker, known as the Operator 4.0, assumes in the work system, and the demands inherent to his/her activity, however, are reconfigured. In this section, according to our second research question (RQ2), we seek to explore how he/she is envisaged. At least until this time, this has been conceptualized through two main visions. Both emphasize the potential of technology, but they have different focuses. That is, either there is the assumption that the human operator is empowered by technology - e.g., “smart operator” through the use of smart technologies, as seen in Romero et al. (2016a) - or that this operator becomes incapable of dealing with all the demands that technology requires and, therefore, he/she will be in need of training and reskilling in order to adapt to the technological change and become efficient in the face of its introduction (e.g., Li, 2022).

While a central role for human workers in the management of these systems is still assumed in the literature, the definitions of the operator appear to be blurred and conceptualized by one common vision, which is the one shared by Romero et al. (2016a,b). According to Romero et al. (2016a), and although they invoke a human-centered approach, operators in I4.0 are defined according to the technological resources in use. They are divided into seven main typologies, which do not necessarily correspond to different workers as more than one of the listed resources can be used in the same work activity: “super-strength operator” (with the resource to exoskeletons); “augmented operator” (with the use of augmented reality), “virtual operator” (supported by virtual reality), “healthy operator” (using smart wearable solutions to measure workers' physical activity), “smart operator” (making use of the available smart technologies), “collaborative operator” (using cobots) and “analytical operator” (using and analyzing Big Data which is collected by the system). Following this perspective, through interaction-based relationships between humans and machines, smart factories are expected to capitalize not only on smart machines' strengths and capabilities, but also empower their operators with new skills and tools (Romero et al., 2016a; Patriarca et al., 2021; Shi et al., 2021). The expectation is for these operators to be in control of work processes and the technology they imply, which these authors flagged contribute to a set of gains for workers in terms of autonomy while developing their own skills too.

Therefore, the Operator 4.0 tends to be depicted as a smart and skilled operator who uses technology according to his/her own needs (Romero et al., 2016b; Kaasinen et al., 2020), or, in other words, “an industrial worker whose cognitive, sensorial, physical and interaction capabilities are enhanced by the close interplay with Industry 4.0 technologies” (Gazzaneo et al., 2020, p. 221). Kaasinen et al. (2020) went further and described smart factories as perfectly fit environments for workers with different skills, abilities, and preferences. However, as mentioned by Longo et al. (2020), this can only be possible if FoF are “designed to embody elicited human values and to illustrate actionable steps that engineers and designers can take in their design projects” (p. 20), as work activity is never disconnected from its socio-historical context, and neither are the workers who perform it.

According to Gajšek et al. (2020), the increase in flexibility will give workers the opportunity to adapt their own working equipment to work demands through their choices when handling these components. For these reasons, in line with what Thun et al. (2019) expressed, I4.0 will change work from being repetitive, low-skilled and physical, to that involving more complex and cognitive tasks, as decentralized decision-making provides a greater degree of autonomy for workers. Although the exact impacts this role reconfiguration will have on workers is still unknown, these reasons are probably why the more cognitive abilities are involved in a task, the more difficult it has been to argue that it can be substituted by technology (Blštáková et al., 2020; Cimini et al., 2020; Golsch and Seegers, 2021). Therefore, as work demands become more complex, these systems may require more “specialization, flexibility, adaptation” increasing qualification requirements and technical skills (Blštáková et al., 2020; Ivaldi et al., 2021). Mark et al. (2019) added that assistance systems can even provide greater chances for the inclusion and support of workers with disabilities, and by including these workers “from the initial planning stage, this potential can be maximized while also making the industrial sector a best practice example of a truly participatory, inclusive field of business” (p. 16).

However, these qualifications and technical skills are not pre-existent to work situations. They are always instigated by work demands in a specific context and developed during action (Teiger and Lacomblez, 2013). Training can contribute to their development but there is no one-size-fits-all workplace learning/training system, as the existing research on digital learning environments is still under development and mainly limited to demonstration-type applications (EU-OSHA, 2018; Engeström, 1999). Also, the literature which provides contributions to workplace training and learning for I4.0 has also placed this issue in debate. On the one hand, technology can create the possibility for new forms of on-the-job training, such as digitalized work directions or virtual training (Hoedt et al., 2020; Chistyakova et al., 2021). On the other hand, training will be a more efficient resource with the integration of real work situations which emerge from the activity, and from the use of technology on a day-to-day basis (e.g., Judon et al., 2019; Galey et al., 2020). The first perspective tends to be the one which is favored in literature.

Longo et al. (2017), by invoking a human-centered approach, offered some valuable contributions in their augmented reality solution for training and providing support for workers during their work. These authors concluded that there was an improvement in performance, but the consideration for aspects such as workers' previous experience or their operational leeway was unclear. Although the study developed by Serras et al. (2020) described a technological support system for industrial maintenance tasks, the fact that workers had no needed expertise to work within it also seems to leave out workers' previous work experience. Ansari et al. (2018) highlighted the importance of “learning strategies,” referring to these as “experience-based, experimental and data-driven strategies enhanced by machine learning and statistical learning methods for both groups of learners, i.e., human or cobots in various competency and autonomy level, respectively” (p. 65). In contrast, even though Hoedt et al. (2020) considered experience and mentioned the support provided should vary accordingly, they used data from sensors to help indicate the workers' competence level and to provide “operator-tailored” support focused on performance and the control of operational cycle time.

While some articles integrated the concept of training and developed solutions for workplace learning, even if still in experimental phases, other authors merely mentioned its importance without further explaining how it would take place (e.g., Fruggiero et al., 2020). But, in fact, and building up on an approach which is anchored in the concrete work activity, when we speak of training, it is not with the aim of contributing to the acceptability of technology or for the transformation of a social representation around it (e.g., fear of job loss). Instead, the aim is to promote the analysis of the representation and the meaning technology has for action, and its enrichment as an experience which is developed from it, as found by Saabye et al. (2020). These authors stressed that, when included, operators can act as active participants (Saabye et al., 2020).

If, on the one hand, the forms of training provided are still to be advanced or sketched out in a more extensive manner, as it is dependent on what kind of knowledge and skills will be needed, other issues in the employment conditions of accessing these jobs will follow. That is, given the objective of reducing monotonous and repetitive tasks, the concern of not having enough qualified workers has arisen alongside it (Blštáková et al., 2020; Iqbal and Ahmad, 2020). Following this perspective, countries which have previously signaled this issue (especially when there is limited access to economic resources for investment) will also originate unequal outcomes when technology is implemented, in addition to the cultural differences which impact their use (Iqbal and Ahmad, 2020). Beyond this point of view, it is critical to explore what is revealed by these technological transformations when looked into using an “activity lens,” namely, the risks and impacts on health.

### Risks and impacts on health: Innovation, but at what cost?

The human-technology relationship takes place in a certain context, under a certain form of work organization. That is, technology is not universal or transferable from one setting to the next without it having implications for the activity developed in it. Therefore, more importantly than identifying the technology-induced risks, they should be understood regarding their specific expression in the contexts in which they emerge (Adriaensen et al., 2019). Until now, there are still gaps in the literature on both these aspects, but particularly on the latter.

Although automation has led to a reduction in manual work, this does not mean physical risks have been fully removed from workplaces. Automated devices could also generate mechanical and electrical hazards, as well as noise, vibration, and chemical or radiation exposure (Leso et al., 2018; Hoyer et al., 2020; Costantino et al., 2021). However, it is the less tangible risks that tend to remain invisible, specifically psychosocial risks (Badri et al., 2018; Bobillier Chaumon et al., 2019; Costantino et al., 2021). A few seem reinforced: irregular work schedules (e.g., 12-h shifts) due to continuous shift working encouraged by automation (Cunha et al., 2020); an increased pressure to work at the speed of the cobot; and a higher level of work supervision made possible through monitoring technologies. These working conditions have negative impacts on physical and mental health, such as musculoskeletal disorders, technostress, or anxiety (Valenduc and Vendramin, 2016; EU-OSHA, 2018; Ghislieri et al., 2018). Moreover, the effects may also have an expression at an infrapathological level (e.g., painfulness; suffering at work), whose evolution can be prevented by monitoring and intervening on their determinant risk factors. Robotics could increase isolated work and reduce the contact with co-workers, which can contribute for the workers' perception of losing control over their professional practices and over the collective criteria used for performing work in quality and health (Bobillier Chaumon et al., 2019). For example, Ivaschenko et al. (2019) developed an augmented reality-supported learning setting, in which supervision by a fellow human worker was substituted by automation, and the impacts of this change for workers' health and wellbeing were not evaluated. If the worker collective assumes a protective role when workers are confronted with work constraints, what resources will they have to develop facing the weakening of the collective activity, when they are expected to interact more with robotic systems than with their co-workers (Blštáková et al., 2020)?

While new technologies can add value to work, they can also constrain the activity by both reinforcing forms of work prescription and reducing operational leeway to be able to use expertise to attain a job well done, in which the worker can recognize him/herself in, and by which he/she seeks to be recognized through. This is crucial for workers' identity, and it is a cornerstone of mental health and wellbeing at work (Bobillier Chaumon et al., 2019). As alluded to by Thun et al. (2019), the workers' autonomy could be at risk with the progress of automation. For instance, in the study of Udayangani et al. (2019), through the development of new automated workstations which control cycle times, performing levels, and the pace of production through alert systems, the workers' operational leeway was not considered. In the aforementioned proposed augmented reality-supported workstation developed by Ivaschenko et al. (2019), the authors intended to prescribe task order, which ultimately ended up restricting the available leeway workers had to develop their activity and ignoring the fact that “workers are not static elements of a complex hierarchical system but are people with knowledge and skills” (May et al., 2015, p. 103). In the literature review developed by Schloegl et al. (2017) reflections on how operators and their variability, differences, professional paths, and other characteristics of the real work activity were also not included in the process of development of assistance systems, even though they used case studies and considered the main tasks developed by the operators.

Many of the studies on I4.0 have focused purely on the technical aspects of the design, ignoring or just partially considering the social relations which support them (Sony and Naik, 2020). As even physical issues such as musculoskeletal disorders are related to the organizational and psychosocial factors of work, their prevention cannot be analyzed separately from the context they are circumscribed to and the relationships supported in it (Coutarel et al., 2022). As a result, the use of these technologies can end up perpetuating negative impacts for workers when these contextual characteristics are overlooked (Barcellini, 2019). In a qualitative study developed by Kadir and Broberg (2020), based on interviews with 35 participants (15 workers and 20 decision-makers) across 10 companies that recently implemented new digital technologies, several factors which impact wellbeing and performance were revealed. Knowledge on how these new work systems function, employer support, job security, or the physical and cognitive load associated with the use of technology were some of these factors (Kadir and Broberg, 2020). What is more, the authors found workers were worried about “causing errors or breaking the new digital technologies” (p. 7), as they knew how expensive they were and were unfamiliar with their use.

Research in the fields of work psychology and activity-centered ergonomics has consistently demonstrated how the development of participatory approaches play an essential role in the exposure to such issues by workers (Béguin and Cerf, 2004; Barcellini et al., 2015; Bobillier Chaumon, 2021). Notwithstanding, many studies are still focused on the potential of technology. For example, in the study carried out by Gualtieri et al. (2020), a manual assembly station was transformed into a collaborative one (with the use of cobots), mainly through the analysis of physical ergonomic assessment, in which the focus was productivity and physical enhancement. Also, Marrella and Mecella (2017), in their “flexible approach for work design,” aimed to tackle unpredictable phenomena at work, through an extensive theoretical framework, but did not consider the real conditions in which activity is developed and its complexity. In another case study developed by Di Carlo et al. (2021), focused on the use of digital twin techniques for safety and maintenance conditions, the authors reported an improvement in the performance of the system, but they also mentioned the emergence of unpredictable difficulties. This can be a result of a deterministic point of view and lack of operator participation. Kolbeinsson et al. (2017), in their analysis on “breakpoints” (i.e., how small interruptions during tasks can improve the quality of assembly operations, considering workers' attention span), still did not consider work activity beyond its technological point of view.

The inclusion of the workers' perspectives during the design processes gives insight into certain types of information exclusive to those who perform work, as their views are rooted in their knowledge of how work is developed on a daily basis (Rangraz and Pareto, 2021). Allied to a frugal innovation model, which promotes sustainable leadership and communication at work (Iqbal et al., 2021), this can contribute to creating a trustworthy relationship between different work actors. Such an approach also provides the opportunity for workers to see how their work is valued and how it contributed to the organization (Saabye et al., 2020; Rangraz and Pareto, 2021).

Despite the foundational aspiration of the I4.0 paradigm of using technical innovation to put the human back at the center (see Saraceno, 2020), human and technical aspects have been perceived asymmetrically, as if the adaptation of the operators to technology was the necessary requirement for work systems to be reliable. Notwithstanding, the importance of the role which the human operator is expected to play in I4.0 contexts seems to be unanimous in literature (e.g., Fantini et al., 2020; Pacaux-Lemoine et al., 2022), recognizing that human intervention remains essential in the work environments which are characterized by the presence of “heterogeneous technologies” (e.g., cobots, exoskeletons, cyber-physical systems) (Barcellini et al., 2021). Aside from having to assure a safe, secure, and efficient interface between these multiple technologies, the operator contributes to the reliability of the work system, for example, by modifying the process configuration when unexpected events occur or a machine breaks down, or by managing work variability and anticipating its potential consequences. However, when the social dimensions associated with innovation projects tend to be kept “in the background,” as noted, the real possibilities, limits, and contradictions of I4.0 technologies are not questioned, nor are their consequences for workers and their professional activities (Barcellini et al., 2021). Bearing this in mind, what is at issue is how to conceive upstream I4.0 innovation projects toward a participatory approach aimed at considering real work activities (in all their complexity and variability), their possible future evolutions triggered by technological change and the possibilities for workers' health preservation. In this context, activity-centered perspectives for design and participatory approaches (Garrigou et al., 1995; Barcellini et al., 2015; Conceição et al., 2017; Broday, 2020; Galey et al., 2020, 2021) become particularly relevant. Garrigou et al. (1995) remind us that such approaches are, first and foremost, *about work*. It is not only the collection of workers' opinion about the transformations to be deployed, but the “construction of a design process in which the designers' knowledge [that tend to be dominant in the design of future work situations] might be confronted with workers' specific knowledge” (Garrigou et al., 1995, p. 312). Such a participation of workers in the process of design future work systems seeks to guarantee that the improvement of future work situations obeys criteria for technical systems' efficiency and for workers' health. And here lies one of the main distinctive attributes of the participatory approach presented in the fields of activity ergonomics and work psychology: the preservation of workers' health and the opportunities for the psychological development of their activity as dimensions of efficiency and performance (Guérin et al., 2021). That means workers' participation in the design process of future work situations should be viewed as “a specific activity” (Garrigou et al., 1995), where one of the central aims, as Lacomblez and Vasconcelos (2009) synthetized, is to reconcile the development of health, performance, and work sustainability.

### Limitations

The current review has been limited by the use of one research database. In addition to this factor, the concepts and technologies explored are, at the time of writing this paper, still under development and under discussion. Therefore, despite the intent of limiting articles which focus on more operationalized technological systems, the use of many of the technologies mentioned throughout this article have been limited to test-type implementations, as noted. This is a relevant consideration as it is through their operationalization in real work contexts, and in a more extended timeline, which technologies can be better conceptualized, and their impacts understood.

## Conclusion

This article provides clues for the understanding of certain risks which can emerge from the application of I4.0 technologies and which can contribute to cautiously shape the future of work instead of following an “undialectical technological determinism” (Howcroft and Taylor, 2014, p. 6). Assuming a techno-centered approach has in fact left little room for debate about the difficulties posed by technology in the real work activity, or the occupational risks and the impacts on health and wellbeing. This is a key issue that has not been sufficiently addressed in literature. The new configurations of human-technology interaction may weaken the human work activity (Badri et al., 2018). On the one hand, the physical risks will not be eliminated from work contexts, and on the other hand, technological change expands the interaction with psychosocial risk factors.

From a more macro point of view, the reconfiguration of the labor market and the forms of work organization brought by I4.0 carries threats to the social sustainability of work. The possible increase in unemployment, digital exclusion, and the unequal distribution of work are some of the threats which have been stressed (Howcroft and Rubery, 2019; Gajšek et al., 2020; Saabye et al., 2020; Saniuk et al., 2020; Rangraz and Pareto, 2021). Saniuk et al. (2020) mentioned some other employment-related risks, such as a mismatch between workers' skills and what these new (or reconfigured) jobs demand.

How can we rehabilitate a human-centered approach to follow and monitor the risks and impacts of these new technologies at work? Criticism on the techno-centered approach highlights the human operator is not an abstract human being, devoid of his/her own and collective history, detached from the dynamic of social relations that underlie his/her job and the conditions where the work activity is performed. Well, the changes induced by I4.0 technologies interact with the outcomes, in each specific situation, of different segmentation vectors in the labor market - particularly, gender, age, job status, or qualification. Therefore, what is at stake is the risk of expanding the existing inequalities instead of diminishing them. The literature has not sufficiently addressed the issue on the new human-machine relations in the context of I4.0 expansion yet. This is a critical moment to explore these blind spots and rethink the work organization and the subsequent inequalities through an intersectionality lens.

Following this viewpoint, a discussion which the articles analyzed in this review seem to overlook is whether the current definitions of the Operator 4.0 see him/her as gender-neutral and, if so, how the changes in work activity and organization affect existing (or create new forms of) gender inequalities, or how gender segmentation interacts with technological development in the I4.0 era.

A study developed by our team in the cork industry (Cunha et al., 2022) showed that gender segregation persisted after the introduction of automation, knowing that this segregation in the division of labor is not independent from the fact that the automatic machines are supported by a legacy of manual activity know-how, which is built by different worker generations. In short, technology interacts with the gender dimension, insofar as it is never gender neutral.

The aging workforce population, expected to increase, also poses a threat to the long-term sustainability of these new work systems (Brozzi et al., 2020). With a large number of older workers expected to remain active for longer, the need for safer work, accessible lifelong training and the employment of older workers become clear needs (Gaudart, 2016). Some authors consider I4.0 to be advantageous, as these systems are supposed to preserve health through the automation of certain physically hazardous, repetitive and monotonous tasks (Brozzi et al., 2020; Agnusdei et al., 2021). However, the demands for learning are more likely to privilege new (possibly younger) workers who are “better equipped to learn” (Badri et al., 2018, p. 407). Plus, the introduction of new technologies cannot actually guarantee that the workers' needs to preserve stability and safety at work will be met (Longo et al., 2020). On the contrary, the increasing work intensification; the constant need to adapt simultaneously to production specificities “that respect neither the same rhythms, nor the same demands, nor the same objectives” (Gaudart, 2016, p. 16); or the irregular work schedules (e.g., Cunha et al., 2020; Rangraz and Pareto, 2021), could challenge the sustainability of these new work systems.

The concept of the Operator 4.0 remains blurry, as does the status of human work in the conceptualization of I4.0 work scenarios, still overshadowed by the expectations of having perfectly fit, healthy, young, gender-neutral, and highly skilled workers, while a multiplicity of risks and possible negative impacts that I4.0 can have for workers emerge.

Technology alone will never be able to live up to the expectations of ideally harmonious, healthy, safe, and more productive than ever work environments (Barcellini, 2020). The human-machine relationship can only be understood through the consideration of the real working conditions and the existing forms of work organization. That is, “thinking about the past and the present is a prerequisite to be able to think about the work of the future” (Barcellini, 2019, p. 12, free translation), since technology will always be shaped by the historical specificities of the work context where it will be used in (Engeström, 1999).

Study cases with workers as the main participants, following both a synchronic and a diachronic analysis of the impacts these work reconfigurations have on health and wellbeing, are thus a necessary step to be addressed in future research, considering that an experience of working with these technologies still has to be developed to provide visibility to emerging risks. Lastly, in the face of the sustainable development goals (United Nations, 2020), and following up on the idea that the Operator 4.0 is not a neutral worker and that work has differentiated impacts on women and men (e.g., Messing and Silverstein, 2009), the gender dimension also ought to be contemplated by future work looking into such impacts for the attainment of healthier (Goal 3), more equal (Goal 5), and more sustainable workplaces (Goal 8). Thus, the pivotal question is how can technology be a driver for the achievement of these objectives?

## Data availability statement

The original contributions presented in the study are included in the article/supplementary material, further inquiries can be directed to the corresponding author/s.

## Author contributions

LC: study conception, design and plan, methodology, analysis and interpretation of results, supervision of research and feedback, and funding acquisition. DS: methodology, data analysis, and visualization. SM: application of the review protocol, data analysis, and data curation. All authors have contributed to writing the original and the final manuscript.

## Funding

This work was supported by the Project OPERATOR: Digital Transformation in Industry with a Focus on the Operator 4.0, co-financed by the ERDF - European Regional Development Fund, through the North Portugal Regional Operational Program and Lisbon Regional Operational Program (NORTE- 01-0247-FEDER-045910 and LISBOA-01-0247-FEDER-045910) - and by the FCT - Portuguese Foundation for Science and Technology, under the MIT Portugal Program (2019 Open Call for Flagship projects).

## Conflict of interest

The authors declare that the research was conducted in the absence of any commercial or financial relationships that could be construed as a potential conflict of interest.

## Publisher's note

All claims expressed in this article are solely those of the authors and do not necessarily represent those of their affiliated organizations, or those of the publisher, the editors and the reviewers. Any product that may be evaluated in this article, or claim that may be made by its manufacturer, is not guaranteed or endorsed by the publisher.
